# Becoming a resident in a high demanded medical specialty: an unequal race? Evidence from the Spanish resident market

**DOI:** 10.1186/s12960-020-00543-2

**Published:** 2021-01-06

**Authors:** Idaira Rodriguez Santana

**Affiliations:** 1 HCD Economics,The Innovation Centre, Keckwick Ln, Daresbury, WA4 4FS United Kingdom; 2 Centre for Health Economics, University of York, York, YO10 5DD United Kingdom

**Keywords:** Medical specialties, Gender gap, Spanish doctors, Occupational segregation, Policy change, I18, I24, J7

## Abstract

**Background:**

Gender occupational segregation in medicine is associated with several undesired consequences such as earnings disparity, shortages of specialists or lower quality of care among others. This paper focuses on the persistent gender gap observed in the most popular specialties of the Spanish resident market. In particular, it explores the role of the specialty allocation system in perpetuating the occupational segregation. For that purpose, this paper studies the effect of a policy change in the ranking system that determines doctors’ specialty choice order. The change increased the competitiveness of the process by increasing the weight of an entry examination from 75% to 90%, in detriment of doctors’ grade point average that decreased from 25% to 10%. Findings from previous literature suggest that that male and female doctors might have reacted differently to the increased competitiveness of the process.

**Methods:**

Data come from administrative records of doctors’ specialty choices for the years 2013 and 2015 and they are used to compute the difference between doctors’ pre and post-change ranking positions. Then, differences in the distribution of rank differences between male and female doctors are tested by means of parametric (*T*-test) and non-parametric (Wilcoxon rank) approaches.

**Results:**

Results show that the policy change has overall favoured male doctors. On average, female doctors lose ranking positions, with respect to the position they would have achieved with the old weights, whilst male doctors gain positions. The differences are more pronounced in the top half of the ranking distribution, meaning that female doctors on average have reduced their probability of accessing the most demanded specialties.

**Conclusions:**

The objective of the policy was the enhancement of the prospects of Spanish-graduate doctors with respect to international graduates by giving more weight to the less prone to bias examination scores. Nonetheless, the change have had the unintended consequence of reducing the probability of female doctors accessing highly demanded specialties and thus exacerbating the gender gap. The allocation system needs revision to make it accountable for the actual role of doctors in society.

## Background

**Fig. 1:**
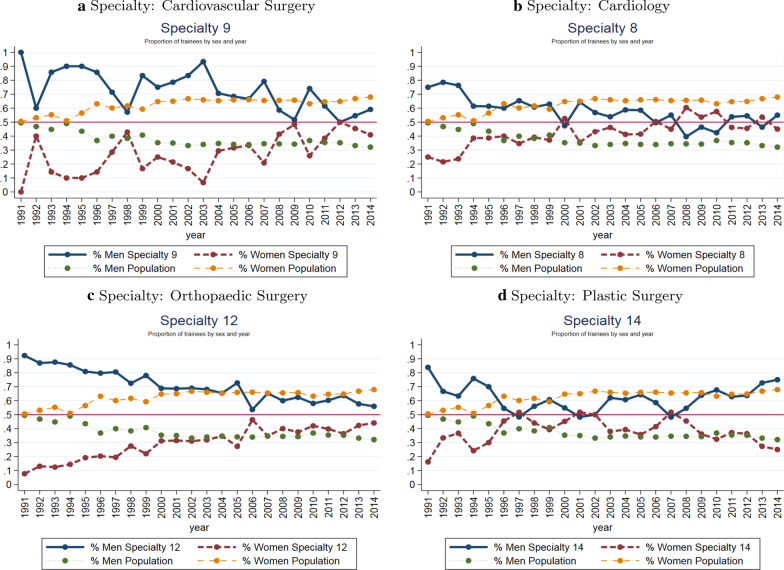
Graphic example of overrepresentation of male junior doctors in four highly demanded specialties overtime. Note: the yellow (green) dotted line represents the total % of first year female (male) junior doctors in a given year. The red line represent the percentage of first year female doctors choosing the specialty that year. If representation was homogeneous the yellow and the red line would overlap; the same would occur with the blue and the green lines that represent the percentage of male doctors choosing that speciality and the total male doctors in that year, respectively

Over the past several decades the medical profession in most developed countries experienced a steady increase in the number of female physicians. Evolving workforce policies, environments and cultural views of gender roles are good examples of the different reasons leading to the feminisation of the medical workforce, a process that have been extensively documented by previous studies [1–5]. In Spain, the share of female doctors registered to practise has risen from 30.4% in 1990 to 51.6% in 2019 [6]. With regard to junior doctors, already in 1991, the percentage of males and females allocated to specialty training had reached equality at: 49.48% and 50.53%, respectively. In 2015, those percentages were 34.31% and 65.69%. These figures illustrate the clear process of the feminisation of the Spanish medical workforce. However, the large increase in the number of women has not been translated to an equal representation of them in each specialty. Females are underrepresented in the high-paid specialties that are in turn the most demanded specialties (see Fig. 1). The traditional explanation is that occupational segregation reflects differences in intrinsic preferences between groups regarding different specialties’ pecuniary and non-pecuniary attributes. Nonetheless, entry barriers to high-demand specialties can be another source of differential attainment. Entry barriers can be *real* such as financial constraints (e.g. oversubscribed specialties often require of higher skills investments), limited access to professional networks, worse employment perspectives for females [7], nepotism [8] and incompatibilities between specialty training schedules and doctors’ personal schedules [9, 10], among others. The barriers can also be *perceived* and therefore closely related to the role of stereotypes and preconceptions affecting doctors’ skills investments [11], perceived gender-based discrimination [12, 13] or to the lack of same-gender role models [10, 14] Alternatively, the occupational segregation might also come from the design of the specialty allocation process as its features might favour one group over the others.

This paper focuses on the analysis of the role of the current Spanish specialty allocation ranking-based system in perpetuating the observed unbalanced specialty outcomes. For that purpose, this paper analyses the effect of a change in the allocation system that took place in the year 2010, which increased the competitiveness of the process, and tests whether it has affected men and women differently, specifically whether it has disadvantaged women as previous studies have documented differences in competitive behaviour between men and women [15–17]. The objective of the change was to ensure the objectivity of the process by increasing the weight of an examination score in detriment to the weight associated with previous attainment in medical undergraduate studies, as regulators see the latter as more prone to biases. I test the differences in the ranking position achieved by male and female doctors that result from the introduction of the new weights by means of a test of equality of means and a non-parametric approach, the Wilcoxon rank-sum test.

The achievement of an equal distribution of doctors across specialties is desirable not only from an equity perspective. There are economic aspects associated with the unequal sorting such as earning disparities between groups, one example being the gender wage gap. It can also lead to large differences in productivity across specialties as there is evidence that females have lower activity rates than male doctors [18] and they tend to work fewer hours than males [19]. Similarly, unequal sorting can lead to large differences in quality of care across specialties as there are documented differences between male and female doctors in communication styles [20], compliance with guidelines [21] and mortality and readmission rates [22, 23]. A more equal distribution of doctors might equalise those differences across specialties and heighten the quality of care in the system as a whole.

### The Spanish allocation system, the policy change and its implications

The allocation process of specialist training positions in Spain is widely known as MIR (‘Médico Interno Residente’) and literally means ‘resident medical intern’. It is organised and regulated centrally by the Ministry of Health [24]. It is a one-sided sequential allocation mechanism where doctors choose their preferred training programme according to their position in a pre-established ranking and specialties play a passive role. Doctors for whom there is no suitable alternative can opt out of the process that year and opt for a position in future calls. The ranking order is a function of doctors’ grade point average in their medical undergraduate studies (GPA) and their score on the MIR examination. The latter is a multiple-choice test that takes place at a national level on the same day and at the same time in different locations across Spain In August 2010, the Spanish Ministry of Health published a list of modifications to the specialty allocation process [25]. The main change was the increase of the importance of the results in the MIR Exam to the detriment of the weight given to the GPA. Specifically, the weight given to the MIR score increased from 75% to 90% and as a result the contribution of the GPA decreased from 25% to 10%. The justification was to ensure the objectivity of the process in the face of an increasing number of non-Spanish medical graduates taking part in it [25]. The results from the MIR examination were viewed as more objective than the GPA, as the latter is considered to be more prone to biases associated with idiosyncrasies from the university (or country) issuing the postgraduate medical certificate.

The outcome of the MIR exam, a one shot test in a highly competitive setting, is the result of a relatively short but very intense period of preparation, defined as *Sprint Effort*, whilst doctor’s GPA is the result of a *Long-Term Effort* [26]. Previous literature suggests that female doctors might be worse off with the new ranking system as it has increased the importance of the highly competitive MIR examination. An economic experiment [15], found that females may be less effective than men in competitive environments, even if they are able to perform similarly in non-competitive ones, due to differences in the ability or propensity to perform in environments where they have to compete against one another. The authors observe that increasing the level of competition improves the performance of men whilst more risk-averse women do not react in same way. Similarly, another experiment [16, 17] found that women are uncomfortable performing in highly competitive settings and as a result choose not to compete and thereby exert less effort than men.

Empirical studies corroborate most of the findings from experimental evidence. A study [27] analysed performance measures in a highly competitive entry exam to a French business school and found that the distribution of exam scores for men had higher means and fatter tails than the distribution for women. However, when analysing long-term measures of performance, defined as *less stressful environments*, women obtained better results. Another empirical study [28] found similar results analysing admission to university in the Czech Republic. If the female doctors’ reaction to the increase in the competitiveness of the MIR process is similar to the observed behaviour of women in this literature, then their ranking outcomes will be lower than the hypothetical ones achieved if the change had not happened.

Worse ranking outcomes for female doctors connect with observed occupational segregation, since most of the male-dominated surgical specialties are in high demand and thus can only be selected by the highest ranking applicants. The combination of high attractiveness and a small supply of training posts leads to a situation where only top ranked students have male-dominated surgical specialties in their choice set. Moreover, taking into account that doctors not only choose their specialty but also location, even marginal changes in the ranking position may put doctors at risk of losing their desired training post. In general, the new weights penalise individuals with a good GPA and reward good performers on the MIR examination.

## Methods

### Data

The MIR Registry is a cross-sectional dataset and comes from doctors’ administrative records held by the Spanish Ministry of Health. It includes a record of doctors’ choices of specialty and training hospital. The data used correspond to the years 2013 and 2015, where 6,348 and 6,015 doctors chose a specialty training post, respectively.

From the MIR Registry, I use the variables *GPA*, the grade point average of medical undergraduate studies that is continuous and ranges from a minimum of 1 to a maximum of 5, and the variable *ES* that refers to MIR examination score. This variable takes only integers and has an upper limit equal to 675.

In order to test differences in the distribution of ranking differences across groups two other variables from the MIR Registry are used: *Women* that takes value one if the doctor is female and zero otherwise and *Spanish* that takes value one when the doctors’ medical undergraduate degree is from a Spanish university and zero otherwise.

### Measuring the change in weights

The variables GPA and ES are used to compute doctors’ pre and post-change ranking positions and the difference between the two, given by the variables RankOld, RankNew and RankDif, respectively. The variable RankDif quantifies the difference in the actual ranking position caused by the change in weights. It results from the subtraction of the ranking position achieved with the new weights, represented by the RankNew, from the position that the individual would have achieved with the pre-change weights represented by variable RankOld and this relationship is shown by expression (1), where $I=\left\{ i\in \mathbb {N}:1\le i\le \bar{I} \right\}$ represents the set of doctors.

1 $$\begin{aligned} \begin{array}{l} \text{RankDif}_{i}= \text{RankOld}_{i}- \text{RankNew}_{i} \\ \text{RankDif}_{i} = F(\text{Total Score}_{\text{old},i})- F(\text{Total Score}_{\text{new},i}) \end{array} \end{aligned}$$

Both RankOld and RankNew result from applying the ranking function to the weighted combination of ES and GPA, as reflected in expression (2). ES and GPA are weighted by fixed values represented by $\alpha$ and $\beta$; $\alpha$ corresponds to the average scores of the top ten MIR examinations, $\left\{ ES_{i(k)}: i(k) \in I,:1\le k \le 10 \right\}$, whilst $\beta$ to the average of the top ten GPAs of the cohort, $\left\{ \text{GPA}_{i(k)}: i(k) \in I,:1\le k \le 10 \right\}$. For each doctor, represented by *i*, we compute the variable RankDif, that equals zero if the doctor keeps the same ranking order with the two different set of weights, i.e. $\text{RankNew}_{i} = \text{RankOld}_{i}$. It is smaller than zero if the doctor is worse off with the new weights, i.e. $\text{RankNew}_{i} < \text{RankOld}_{i}$, and greater than zero if the doctor is better off, i.e. $\text{RankNew}_{i} > \text{RankOld}_{i}$.

2 $$\begin{aligned} \begin{array}{l} \text{Total score}_{\text{old},i} = \dfrac{75}{\alpha }ES_{i} + \dfrac{25}{\beta }\text{GPA}_{i} \\ \\ \text{Total score}_{\text{new},i} = \dfrac{90}{\alpha }ES_{i} + \dfrac{10}{\beta }GPA_{i}\\ \end{array} \end{aligned}$$

To test if the change in weights affect men and women differently, we perform a test of equality of means to the variable RankDif. I assume a common variance for the individuals of the same gender, but allow the variance to be different between men (*m*) and women (*w*). The test for equality of means is given by expression (3), where $\mu$ represents the mean and *s* the standard deviation of the variable RankDif, $N_{m}$ and $N_{w}$ the number of male and female doctors, and *t* follows a Student’s *t* distribution:

3 $$\begin{aligned} t= \dfrac{\mu _{m} - \mu _{w}}{\sqrt{\dfrac{s_{m}^{2}}{N_{m}} + \dfrac{s_{w}^{2}}{N_{w}}}}. \end{aligned}$$

Moreover, as RankDif only takes integers and its distribution might cast doubt on its normality, I also apply a non-parametric approach, the Wilcoxon rank-sum test [29, 30] that tests null the hypothesis that two samples (i.e. the samples for male and female doctors) are from populations with the same distribution. The construction of Wilcoxon statistic *T* involves jointly ranking the values of $\text{RankDif}_{i}$ from smallest to largest of both men and women, whose sample sizes are given by $n_{m}$ and $n_{w}$, respectively. The smallest $\text{RankDif}_{i}$ is given the value 1 whilst the largest is given the value $n=n_{m} + n_{w}$. The second step is to sum the ranking numbers associated with the observations of the group that we denote as first, in this case the one comprised of male doctors, as given by (4):

4 $$\begin{aligned} T=\sum _{i=1}^{n_{m}} R_{1i}. \end{aligned}$$

As the sample size is sufficiently large, we can use the normal approximation given by expression (5):

5 $$\begin{aligned} z= \dfrac{T- E[T]}{\sqrt{Var(T)}}, \end{aligned}$$

where $E[T]=\dfrac{n_{m}(n+1)}{2}$, $Var[T]=\dfrac{n_{m}n_{w}}{n}s^{2}$ and *s* is the standard deviation of the combined ranking. Finally, we compute the probability of observing that $\text{RankDif}_{\text{Men}}> \text{RankDif}_{\text{Women}}$ for any two random observations, and this is given by expression (6):

6 $$\begin{aligned} Pr(\text{RankDif}_{\text{Men}}> \text{RankDif}_{\text{Women}})= \dfrac{2T - n_{m}(n_{m} +1 )}{2n_{m}n_{w}}. \end{aligned}$$

Individuals are sorted according to their actual ranking position (RankNew) and divided into 13 groups. The top group encompasses the top 499 achievers and the bottom group the doctors who chose a specialty training post in the position 6000 or below. The objective is to analyse if the change in weights affects top, middle and bottom ranked students differently. Results of the RankDif for the reduced sample of students who graduated from a Spanish university are also shown to test whether the policy fulfils its original purpose.

### Limitations

Doctors for whom there is no suitable alternative left at the moment of the choice can opt out of the process that year and they are not included in the MIR Registry. Therefore, in this paper I use the ranking position of the actual choice rather than the original position that also includes opt outs. Differences between the two measures are minimal and should not affect the results.

The variable ES is only available for years 2013 and 2015; both years are from the period post-change in weights, and therefore we are not able to test how the change would have modified the ranking of the doctors who chose a specialty before the change was implemented. Moreover, the ideal assessment of the effect of the change in the ranking system would require knowledge of how the same individual would behave in the pre and post-weights change periods. We would require a counterfactual observation for each individual indicating what the outcome would have been if the change would have not taken place. Hence, to assess the effect of the change we need to assume that the new weights have neither affected medical students’ GPAs nor MIR examination scores. This seems a reasonable assumption for GPA, as it is a long-term measure that combines the effort of the student throughout the medical undergraduate studies and it seems unlikely that students would modify their long-term strategy half-way through their bachelor’s degree. Most students do not decide what specialties they would like to practise until the final stage of their medical studies [31] and, therefore, GPA may be a fair representation of their best effort in order to keep all options open. However, it is likely that a medical student in the face of the increased importance of the MIR examination would respond to the change by exerting more effort in the exam preparation. It is well-known that students adapt their exam preparation effort to their desired specialty [32] and that the increase in competition could have increased the effort in preparation for all doctors but that the effect might be larger for men than women [16, 17]. Hence, by failing to include a counterfactual observation in the analysis we could be underestimating the effect of the change in the weights.

## Results

### Results MIR 2013

Table 1 shows the descriptive statistics for the variable RankDif by gender, reports the differences in means represented by $\Delta \text{RankDif}$, the results of the *t*-test and the Wilcoxon rank-sum test for the population of doctors who chose their specialty in the year 2013. There were 6348 doctors and from those 2106 were men and 4242 women. On average, male doctors gain 11.4 positions whilst female doctors lose 5.7 positions. Therefore, $\Delta \text{RankDif}$ indicates a difference of 17.1 positions between the two groups and this is statistically significant with a p-value $p<$0.05. The results from the Wilcoxon test show that the probability of observing $\text{RankDif}_{\text{M}} > \text{RankDif}_{\text{W}}$ is 0.53, suggesting that the distribution of the variable RankDif is different for men and women.

The distribution of male and female doctors in the different ranking intervals is far from being equal and the largest difference can be found in the group of top achievers ($<500$), where the share of male doctors is 10% whilst the share of female doctors is only 6.8%. In addition, $\Delta \text{RankDif}$ for the group of top achievers is positive, equalling 31.3 and statistically significant ($p<$0.01). The breakdown of RankDif for that group suggests that both male (36.5) and female (5.2) doctors gain positions with the introduction of new weights, but that the improvement is bigger for male doctors.

**Fig. 2:**
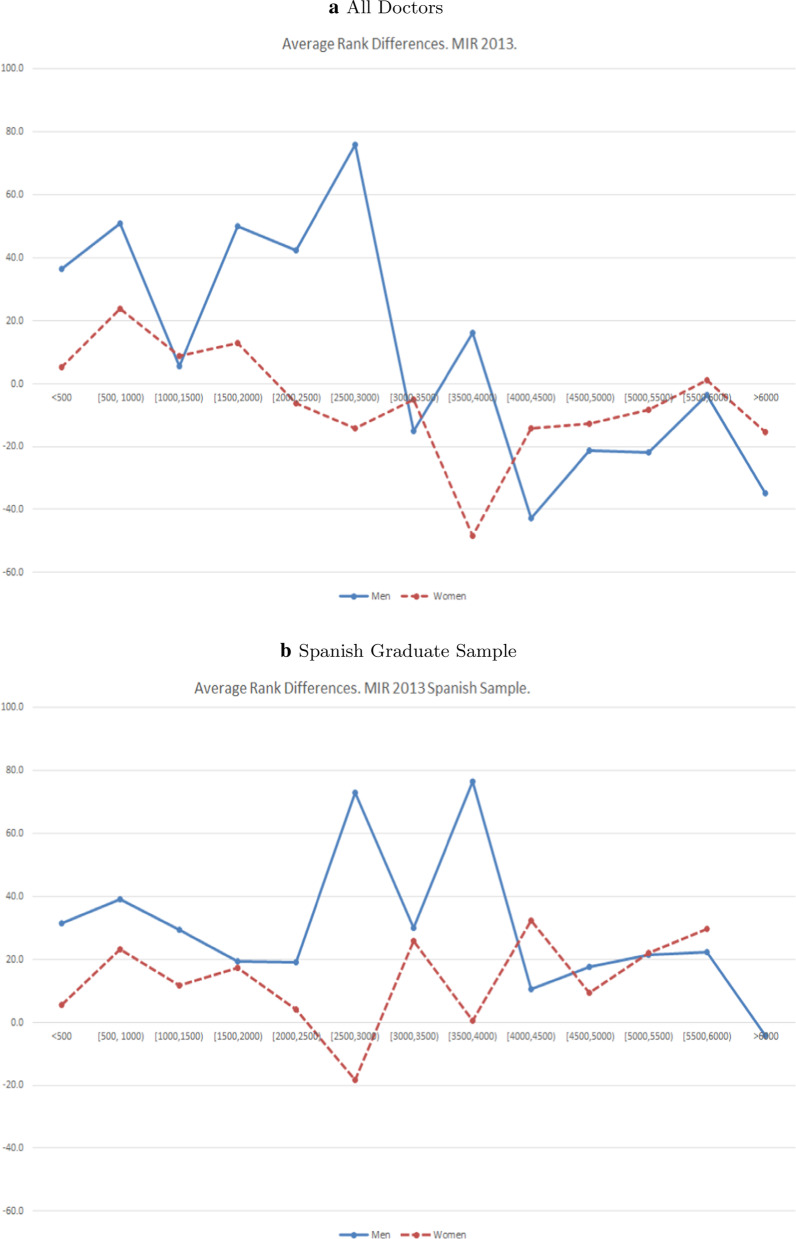
Average RankDif by gender. MIR 2013

There is a clear gender gap in the distribution of the RankDif, as shown by Fig. 2. All the statistically significant $\Delta \text{RankDif}$ are positive, and for all intervals at the top end of the ranking, i.e. those doctors who ranked in the first 3000 positions, differences are always positive, meaning that a typical top achieving male doctor gains, on average, more positions than the equivalent top achieving female doctor. The results of the Wilcoxon test are similar to the results observed for the test of equality of means.

**Table 1:** Differences in ranking position by gender: MIR 2013

MIR 2013	Men				Women				Student’s *t*-test		Wilcoxon rank-sum	
Ranking	N	Share (%)	$\text{RankDif}_{M}$	SD	N	Share (%)	$\text{RankDif}_{W}$	SD	$\Delta \text{RankDif}$	*t*	$PR^{a}$	*z*
$<500$	211	10.0	36.5	103.7	288	6.8	5.2	104.1	31.29***	3.32	0.582***	3.15
[500, 1000)	166	7.9	50.8	195.5	334	7.9	23.7	173.6	27.08	1.51	0.536	1.315
[1000, 1500)	162	7.7	5.4	235.4	338	8.0	8.8	220.8	− 3.38	− 0.15	0.502	0.059
[1500, 2000)	168	8.0%	50.0	278.2	332	7.8	12.9	256.5	37.09	1.45	0.541	1.498
[2000, 2500)	149	7.1	42.4	337.2	351	8.3	− 6.3	268.3	48.76	1.57	0.565**	2.31
[2500, 3000)	150	7.1	75.9	290.8	350	8.3	− 14.3	317.7	90.20***	3.09	0.579***	2.799
[3000, 3500)	173	8.2	− 15.2	341.7	327	7.7	− 5.2	295.2	− 10.01	− 0.33	0.503	0.105
[3500, 4000)	154	7.3	16.2	290.5	346	8.2	− 48.3	294.8	64.52**	2.28	0.575***	2.674
[4000, 4500)	165	7.8	− 42.7	287.8	335	7.9	− 14.3	241.6	− 28.43	− 1.09	0.489	− 0.413
[4500, 5000)	170	8.1	− 21.2	246.8	330	7.8	− 12.7	201.2	− 8.51	− 0.39	0.52	0.751
[5000, 5500)	154	7.3	− 22.1	160.1	346	8.2	− 8.5	151.1	− 13.58	− 0.89	0.48	− 0.72
[5500, 6000)	160	7.6	− 3.7	129.2	340	8.0	1.0	121.2	− 4.71	− 0.39	0.496	− 0.158
$\ge 6000$	124	5.9	− 34.8	117.7	225	5.3	− 15.4	85.3	− 19.44	− 1.62	0.468	− 0.982
Total	2106	100	11.4	246.3	4242	100	− 5.7	227.1	17.06***	2.67	0.526***	3.394

P-values:***p < 0.01, **p < 0.05, *p < 0.1

$$^{a}$$ $$\text{PR}=Pr(\text{RankDif}_{M}> \text{RankDif}_{W})$$

### Results MIR 2013: Spanish Graduate Sample

Table 2 shows results for the sample of doctors who graduated from a Spanish university. Also in this sub-sample, male doctors are over-represented in the two top groups of high achievers $(<500)$ and [500, 1000). The $\Delta \text{RankDif}$ for the entire sample is positive and equal to 17.8 ($p<$0.01). In this sample, both male and female doctors are better off with the introduction of the new weights, however the magnitude of the gain is greater for men, who gain on average 31 positions, than for women, who *only* gain 13.2. The Wilcoxon test confirms the previous result and shows that the probability of observing $\text{RankDif}_{\text{M}} > \text{RankDif}_{\text{W}}$ is 0.53 ($p<$0.01).

**Table 2:** Differences in ranking position by gender: MIR 2013-Spanish Graduate Sample

MIR 2013	Men				Women				Student’s *t*-test		Wilcoxon rank-sum	
Ranking	N	Share (%)	$\text{RankDif}_{M}$	SD	N	Share (%)	$\text{RankDif}_{W}$	SD	$\Delta \text{RankDif}$	*t*	$PR^{a}$	*z*
$<500$	184	13.8	31.5	94.2	273	8.0	5.5	101.8	26.02***	2.803	0.575***	2.707
[500, 1000)	134	10.0	39.2	176.5	304	8.9	23.2	174.2	15.92	0.873	0.521	0.699
[1000, 1500)	115	8.6	29.4	203.8	293	8.5	11.6	210.3	17.77	0.785	0.529	0.921
[1500, 2000)	109	8.2	19.2	255.1	288	8.4	17.1	241.3	2.11*	0.075	0.507	0.21
[2000, 2500)	96	7.2	18.9	322.5	294	8.6	4.1	251.1	14.76	0.410	0.525	0.741
[2500, 3000)	93	7.0	73.0	243.7	271	7.9	− 18.4	279.0	91.42***	3.005	0.594***	2.706
[3000, 3500)	90	6.7	30.0	268.6	260	7.6	25.8	245.5	4.19	0.131	0.516	0.444
[3500, 4000)	99	7.4	76.5	225.8	275	8.0	0.4	248.0	76.05***	2.798	0.600***	2.937
[4000, 4500)	95	7.1	10.5	255.6	263	7.7	32.1	171.1	− 21.60	− 0.764	0.516	0.457
[4500, 5000)	88	6.6	17.6	201.4	252	7.4	9.3	156.8	8.24	0.349	0.555	1.536
[5000, 5500)	81	6.1	21.3	112.0	275	8.0	22.0	102.8	− 0.76	− 0.055	0.509	0.256
[5500, 6000)	88	6.6	22.3	99.6	241	7.0	29.6	84.0	− 7.32	− 0.614	0.489	− 0.299
$\ge 6000$	63	4.7	− 4.3	68.9	138	4.0	8.9	56.5	− 13.16	− 1.326	0.442	− 1.321
Total	1335	100.	31.0	206.1	427	100	13.2	197.1	17.80***	2.709	0.530***	3.268

P-values: ***p < 0.01, **p < 0.05, *p < 0.1

$$^{a}$$ $$\text{PR}=Pr(\text{RankDif}_{M}> \text{RankDif}_{W})$$

In general, the results are similar to the ones reported in Table 1, however the magnitude of the variable $\Delta \text{RankDif}$ is smaller for the top achievers and larger for doctors situated in the central positions of the ranking distribution as shown by Fig. 2.

### Results MIR 2015

Table 3 shows the results for the sample of doctors who took part in the MIR in 2015. Distribution of doctors by gender is similar to the observed in 2013: 2,064 are male and 3,951 female. On average, male doctors gained 10.4 positions whilst female doctors lost 5.4 positions. and results in a difference of 15.7 positions ($p<$0.05). The Wilcoxon test shows that the probability of observing $\text{RankDif}_{M} > \text{RankDif}_{W}$ is 0.52 ($p<$0.01), indicating that the distribution of the variable RankDif is different for men and women.

**Table 3:** Differences in ranking position by gender: MIR 2015

MIR 2015	Men				Women				Student’s *t*-test		Wilcoxon rank-sum	
Ranking	N	Share (%)	$\text{RankDif}_{M}$	SD	N	Share (%)	$\text{RankDif}_{W}$	SD	$\Delta \text{RankDif}$	*t*	$PR^{a}$	*z*
$<500$	234	11.3	18.2	88.2	265	6.7	23.4	98.6	− 5.18	− 0.619	0.509	0.354
[500, 1000)	203	9.8	71.4	220.1	297	7.5	28.3	192.9	43.15**	2.262	0.560**	2.264
[1000, 1500)	188	9.1	51.2	280.9	312	7.9	10.9	258.5	40.30	1.600	0.542	1.556
[1500, 2000)	158	7.7	51.2	297.9	342	8.7	− 13.6	289.0	64.77**	2.282	0.563**	2.26
[2000, 2500)	188	9.1	7.7	300.6	312	7.9	− 22.1	289.8	29.75	1.087	0.536	1.345
[2500, 3000)	164	7.9%	42.9	308.0	336	8.5	11.4	301.9	31.48	1.080	0.532	1.162
[3000, 3500)	163	7.9	− 21.6	376.0	337	8.5	− 17.2	324.7	− 4.35	− 0.127	0.511	0.405
[3500, 4000)	146	7.1	− 22.3	288.2	354	9.0	− 38.8	259.4	16.51	0.599	0.526	0.916
[4000, 4500)	132	6.4	− 19.9	260.8	368	9.3	− 17.7	215.6	− 2.22	− 0.088	0.528	0.956
[4500, 5000)	154	7.5	− 32.5	222.7	346	8.	− 5.3	173.1	− 27.22	− 1.346	0.468	− 1.134
[5000, 5500)	140	6.8	− 33.8	176.3	360	9.1	− 3.9	132.2	− 29.92*	− 1.819	0.457	− 1.483
[5500, 6000)	186	9.0	− 23.1	130.4	314	7.9	− 8.1	94.7	− 15.01	− 1.371	0.481	− 0.729
$\ge 6000$	8	0.4	− 3.8	5.9	8	0.2	− 0.3	6.3	− 3.50	− 1.145	0.336	− 1.113
Total	2064	100.0	10.4	255.1	3951	100.0	− 5.4	234.1	15.86**	2.353	0.522***	2.847

P-values: ***p < 0.01, **p < 0.05, *p < 0.1

$$^{a}$$ $$\text{PR}=Pr(\text{RankDif}_{M}> \text{RankDif}_{W})$$

**Fig. 3:**
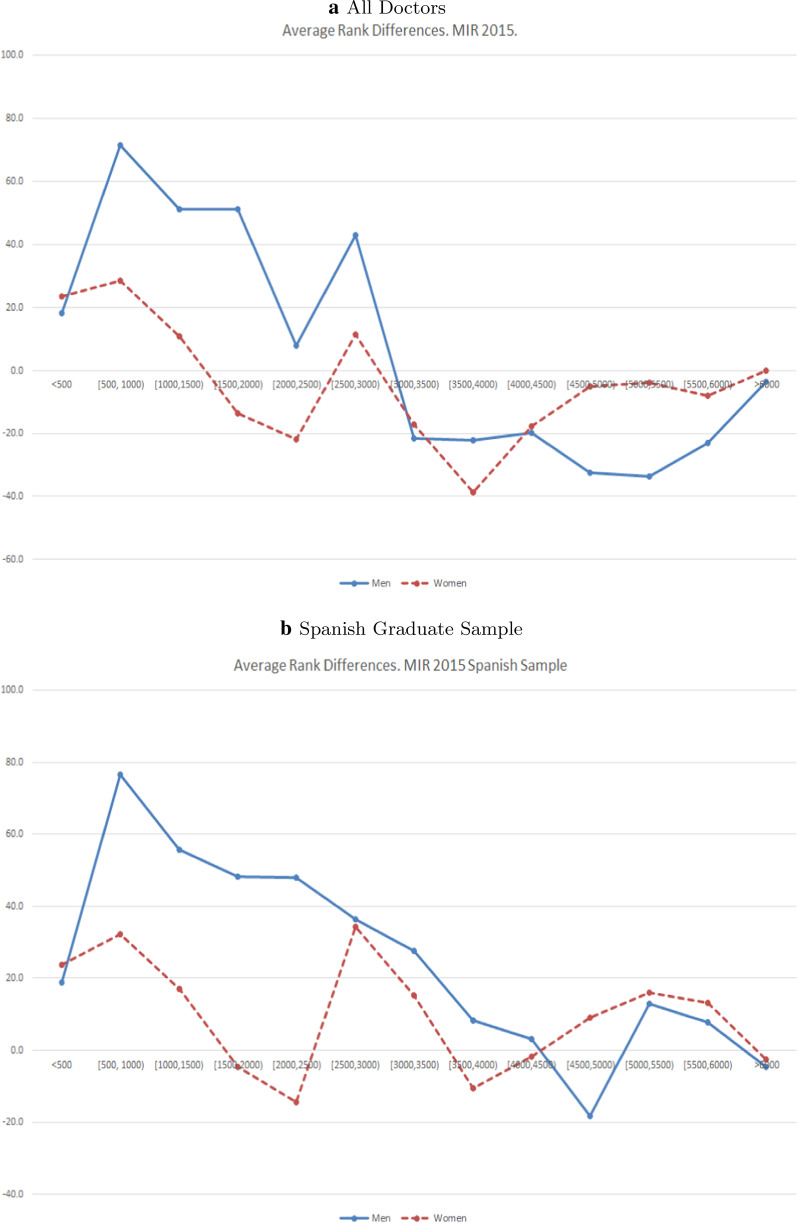
Average RankDif by gender. MIR 2015

In 2015, women from the top group ($<500$) gained, on average, more positions than men with the introduction of the new weights ($\Delta \text{RankDif}$ < 0). However, the negative difference fails to be statistically significant. $\Delta \text{RankDif}$ is statistically significant for the groups [500, 1000) and [1500, 2000) and equal to 43.2 and 64.8, respectively. Figure 3 shows the distribution of RankDif for the MIR 2015.

### Results MIR 2015: Spanish Graduate Sample

Results in Table 4 show that the overall $\Delta \text{RankDif}$ is positive and equal to 19.2 (*p* < 0.01) meaning that both male and female doctors are better off with the introduction of the new weights. However, the magnitude of the gain is greater for men who gain on average 29.5 positions, whilst women gain 10.3. The differences in the distribution of RankDif are confirmed by the Wilcoxon test, as the probability of observing $\text{RankDif}_{\text{M}} > \text{RankDif}_{\text{W}}$ is 0.53 (p<0.01). The breakdown of $\Delta \text{RankDif}$ by ranking intervals present similar results to those observed in Table 3 for the complete sample.

**Table 4:** Differences in ranking position by gender: MIR 2015-Spanish Graduate Sample

MIR 2015	Men				Women				Student’s *t*-test		Wilcoxon rank-sum	
Ranking	N	Share (%)	$\text{RankDif}_{M}$	SD	N	Share (%)	$\text{RankDif}_{W}$	SD	$\Delta \text{RankDif}$	*t*	$PR^{a}$	*z*
$<500$	224	13.5	18.9	89.0	259	7.3	23.8	98.4	− 4.99	− 0.585	0.510	0.388
[500, 1000)	177	10.6	76.6	211.5	278	7.8	32.3	189.7	44.32**	2.268	0.564**	2.316
[1000, 1500)	160	9.6	55.8	261.0	294	8.2	17.0	258.2	38.82	1.520	0.545	1.583
[1500, 2000)	131	7.9	48.3	277.9	317	8.9	− 4.7	281.6	52.99*	1.829	0.543	1.446
[2000, 2500)	144	8.7	48.1	261.2	280	7.8	− 14.4	265.5	62.43**	2.318	0.567**	2.254
[2500, 3000)	129	7.8	36.3	297.2	308	8.6	34.3	279.9	2.00	0.065	0.513	0.414
[3000, 3500)	123	7.4	27.6	363.0	301	8.4	15.1	294.3	12.42	0.337	0.535	1.144
[3500, 4000)	112	6.7	8.2	261.0	317	8.9	− 10.6	234.7	18.84	0.674	0.533	1.038
[4000, 4500)	109	6.6	3.2	245.8	336	9.4	− 1.7	183.3	4.94	0.193	0.546	1.447
[4500, 5000)	129	7.8	− 18.2	218.5	313	8.8	9.0	149.2	− 27.20	− 1.295	0.475	− 0.815
[5000, 5500)	101	6.1	12.9	93.3	319	8.9	16.1	101.7	− 3.21	− 0.295	0.477	− 0.707
[5500, 6000)	118	7.1	7.7	73.4	244	6.8	13.2	72.1	− 5.55	− 0.678	0.481	− 0.574
$>6000$	7	0.4	− 4.7	5.6	6	0.2	− 2.7	5.2	− 2.05	− 0.678	0.393	− 0.649
Total	1664	100	29.5	233.3	3572	100.0	10.3	216.7	19.18***	2.832	0.528***	3.278

P-values: ***p < 0.01, **p < 0.05, *p < 0.1

$$^{a}$$ $$\text{PR}=Pr(\text{RankDif}_{M}> \text{RankDif}_{W})$$

## Discussion

The results show that the policy change that increased the weight of the MIR examination, to the detriment of the weight associated with the grade point average, has overall favoured male doctors. On average, female doctors lose ranking positions, with respect to the position they would have achieved with the old weights, whilst male doctors gain positions. The differences are statistically significant. The results for the reduced sample of Spanish graduates specifically show that, on average, both male and female doctors are better off after the change, however the magnitude of the gain is substantially smaller for the female doctors. Similarly, the breakdown of the ranking differences by ranking position shows that top achievers, both men and women, are better off than bottom achievers after the change; however, the magnitude of the gain is again smaller for female top achievers than for male top achievers.

The results from the bottom half of the ranking distribution need to be interpreted with caution, as the number of doctors opting out of the MIR process increases when the number of training positions reduces. Hence, the discrepancies between the original ranking and the actual choice order are larger here than among the top achievers. I expect the proportion of male doctors dropping out of the process to be larger than the proportion of females, as historically male doctors have shown stronger preference for the most demanded specialties and, therefore, bottom achieving female doctors who historically have shown preference for the less demanded specialties such as general practice, might be over-represented at the bottom of the ranking distribution. If the latter is confirmed and we only observe fewer and/or worse-achiever males, it would (partially) explain the observed change in trends at the bottom half of the ranking distribution.

The results corroborate the findings from experimental economics which conclude that women are more reluctant to engage in competitive interactions and as the competitiveness of an environment increases, the performance and participation of men increase relative to women[33]. The reluctance is usually explained by women’s higher levels of risk aversion and also with an excess entry in the competition level of men due to their overconfidence [34]. These differences in risk and confidence are consistent with the observed strategies taken by female and male doctors on the MIR examination. A Spanish study [35] constructed a measure of risk taken in the MIR examination finding that males take greater risk than female doctors, and that translates to better results for the top achievers and worse results for the male doctors at the bottom end of the ranking distribution. The observed behaviour by [35] is very similar to the results for the distribution of $\Delta \text{RankDif}$. The differences in ranking are, on average, positive, favouring male doctors in the top half of the ranking distribution; specifically, male top achievers who might have taken more *risk* and where their behaviour entails an increase in their MIR score. By contrast, male doctors in the bottom half of the ranking distribution might also have taken more *risk* in responding to the test, however that group presents a lower success rate, as they are more likely to have incorrect answers, and for them the extra risk taken translates to a negative $\Delta \text{RankDif}$.

## Conclusion

This paper explores one of the sources of the occupational gender segregation in the Spanish medical workforce, and the findings suggest that a policy change have had the unintended consequence of reducing the probability of female doctors accessing high-demand specialties. The original design of the MIR allocation system and the posterior change in weights were motivated to ensure the reliability and transparency of the process, and to avoid favouritism [36]. The Spanish specialty allocation system is based on the principle of vertical equity, as it permits the most productive candidates to have the highest priority in choosing a training programme, using ranking position as a proxy for doctors’ productivity [37]. Nonetheless, the findings suggest that ranking position might not be a fair proxy of productivity, as there is a clear differential in attainment on the MIR examination results between male and female doctors. The MIR examination only evaluates medical knowledge, by means of a restrictive multiple-choice test, does not value other important aspects such as communication, empathy or professionalism [36], and neglects the importance of having real vocation for the chosen specialty [38]. For those other non-valued aspects there is evidence of females outperforming male doctors. Using data from the United Kingdom, [39] and [40] found that women were more likely to outperform men in clinical skill examinations that take place during the specialty training residency. Moreover, there is evidence that females are more likely to adhere to clinical guidelines [21] and that female doctors provide preventive care more often than male doctors [41].

Therefore, there is a need for an in-depth revision of the functioning of the process in order to adapt it to the demographic composition of new cohorts of doctors, to the actual role of doctors in society, and to make it accountable for the other competences that are valued in the practise of modern medicine.

## Data Availability

The data that support the findings of this study are available from The Spanish Ministry of Health but restrictions apply to the availability of these data, which were used under license for the current study, and so are not publicly available.

## Abbreviations

GPA: Grade point average
MIR: Medico Interno Residente
